# “They turn to you first for everything”: insights into midwives’ perspectives of providing physical activity advice and guidance to pregnant women

**DOI:** 10.1186/s12884-019-2607-x

**Published:** 2019-12-03

**Authors:** Marlize De Vivo, Hayley Mills

**Affiliations:** Section of Sport and Exercise Sciences, School of Human and Life Sciences, Canterbury Christ Church University, Canterbury, UK

**Keywords:** Midwife, Pregnancy, Maternal, Physical activity, Exercise, Qualitative, Thematic analysis, England

## Abstract

**Background:**

The antenatal period is associated with a decline in physical activity among women. Midwives are viewed central to the dissemination of information during pregnancy, however, there is little research relating to their promotion of physical activity. The purpose of this study was to gain insight into midwives’ perspectives of providing physical activity advice and guidance to pregnant women

**Methods:**

Community midwives (N = 10) from ten randomly selected antenatal clinics in England took part in semi-structured interviews which were audio recorded, transcribed and analysed thematically

**Results:**

In relation to perceived role and responsibilities in providing physical activity advice and guidance, midwives emphasised the extent to which their profession has evolved, the perceived burden of responsibility, and the tick box approach to physical activity discussion. Midwives identified a lack of training, knowledge, confidence, time, resources, and perceptions of vulnerability as barriers to effective physical activity promotion. Despite these issues midwives proposed eight opportunities to facilitate pregnant women’s physical activity engagement: (1) recognising and addressing barriers in the uptake and maintenance of physical activities, (2) professional development, (3) inter-professional collaboration, (4) communicating effectively through simple, reliable resources, (5) improved access, availability, and awareness of suitable activities in the local community, (6) encouraging a support network, (7) “selling” physical activity by challenging misconceptions and focusing on benefits, and (8) suitable motivation, incentives and reward.

**Conclusions:**

These opportunities identified by midwives provide foundations from which improvements in practice can result. Whilst midwives are ideally placed to promote physical activity as part of a healthy pregnancy, this is likely to be most effective as part of a wider network of practitioners that share consistent and confident messages regarding physical activity engagement. This notion has far reaching implications for practice, policy, research and the normalisation of active pregnancies in the wider population.

## Background

It is recognised that the lifestyle choices made by a pregnant woman can have profound effects on the health of both mother and baby and that these can extend beyond gestation and birth [1, 2]. Pregnancy is associated with a decline in physical activity among women [3], and at the same time is viewed as a window of opportunity to address lifestyle issues [4]. It is therefore not surprising that midwives are regarded as being ideally placed to advise on diet and activity changes during pregnancy [5]. However, there is little research relating to the promotion of physical activity by midwives, with the current focus arguably on the management of pregnancy weight gain and maternal obesity [6]. Women with overweight and obesity have access to specialist maternity care pathways [7], there is a lack of pregnancy specific and consistent physical activity guidance in the United Kingdom (UK) for normal uncomplicated pregnancies [8]. Whilst the benefits of an active lifestyle is recognised in various professional documents - such as Statement 4 by the Royal College of Obstetricians and Gynaecologists (RCOG) [9] - at the time of this study there were no national physical activity recommendations for pregnant women. In a topical call for research and guidance, Reid, Smith, Calderwood, and Foster [8] suggest that the views of healthcare professionals in relation to their role in the promotion of physical activity should become a research priority. Therefore, in recognition of midwives’ central role in disseminating information during pregnancy, the primary purpose of this study was to gain insights into midwives’ perspectives of providing physical activity advice and guidance to pregnant women. Three research questions were developed for this purpose:

i. what are midwives perceived roles and responsibilities in providing physical activity advice and guidance to pregnant women;
ii. what are the barriers perceived by midwives in providing effective physical activity advice and guidance to pregnant women; and
iii. do midwives perceive any opportunities in changing pregnant women’s physical activity behaviour?

## Methods

### Design

This qualitative study involved a semi-structured interview approach and formed part of a multiphase mixed methods research project with the overriding aim of understanding and predicting the physical activity behaviour of pregnant women. Sixty-two National Health Service (NHS) antenatal clinics across East Kent were identified and notified in writing that ethical approval and permission to carry out the study had been granted and that the antenatal clinics hosted at these locations could be selected at random to form part of the various phases of the study. Following notification, each location was assigned a number and selected through a computer-generated randomization procedure. Ten locations were randomly selected as the recruitment sites for this study.

### Participants

Midwives were eligible for participation if they were at least 18 years of age, proficient in the English language, practising as a community midwife, and had been qualified for more than 1 year. Of the 19 midwives who were approached, 10 agreed and took part in the study. Participants had a mean age of 50.8 years (SD = 8.44) with midwifery experience ranging from 5 to 37 years (M = 16.50, SD = 11.55). Of the six participants who reported their employment status, four indicated that they were employed full-time. All midwives had two or more children of their own.

### Procedure

The first author explained the study and provided potential participants with an information sheet, consent form and demographics questionnaire. Midwives who agreed to participate were asked to return their signed consent form and completed demographics questionnaire to the researcher and were subsequently contacted by either e-mail or phone where a date, time, and venue for the interviews to take place was agreed.

The first author drafted an interview guide informed by previous research and the data produced during earlier phases of the mixed methods research project [10]. Minor revisions were made to the semi-structured interview schedule following a pilot interview with a practicing community midwife and discussion with the second author (Additional file 1).

Face-to-face interviews took place during March to July 2015 with each interview lasting 31 min on average (range 20–74 min). Except for one, all interviews took place at participants’ place of work. All interviews were audio recorded and transcribed verbatim by the first author. A copy of the transcript was subsequently made available to each participant, providing them with the opportunity to offer further comment or clarification on the issues raised.

### Analysis

A combination of inductive and deductive approaches were used to produce a thematic description of the data. Specifically, the research questions served as an a priori template for the initial deductive management of data. Themes and subthemes were then inductively identified through a six-stage process of thematic analysis involving: (1) data familiarization, (2) data coding, (3) identification of themes, (4) revision of themes, (5) defining and naming of themes, and (6) writing up [11].

Clarke and Braun [11] recommend a minimum sample of six participants when analysing interviews with the focus on identifying patterned meaning across cases rather than idiographic meaning within cases. Data saturation is, however, also suggested to be a function of the interview structure and content, and the homogenous nature of the sample, specifically, the more similarities and consistencies shared between participants, the sooner data saturation will be achieved [12]. In this context, it should be noted that the midwives participating in this study represented such a sample and that data saturation occurred at the recommended minimum sample level.

Following review of the identified themes with the second author, the findings were considered and discussed in the context of the National Maternity Review [13]. In doing so, the authors demonstrate how the promotion of physical activity during pregnancy fits within the five-year plan for improving maternity outcomes and services in England and links with wider debates about the challenges within maternity care.

Issues of trustworthiness were addressed through strategies such as member checking, source triangulation and author agreement which attests to the rigour of the overall approach [14]. Furthermore, when presenting the results, selected examples of participant quotes (i.e. raw data) are used to illustrate themes. To preserve anonymity, participants were assigned with pseudonyms.

## Results

### What are midwives perceived roles and responsibilities in providing physical activity advice and guidance to pregnant women?

It is apparent that despite the varied and complex scope of the profession, midwives still observe a role and responsibility in advocating regular physical activity as part of a healthy pregnancy, however, there are various factors influencing their daily practice. Three main themes represent these perceptions (see Table 1).

**Table 1:** The midwifery profession has evolved with consequences for current practice

Raw data example	Subtheme	Theme
“The main challenges are time and my case load is big. Umm, I only work four days a week but I’ve almost got more than a full-time case load, so it’s trying to fit everything in, umm, it’s difficult. The demands of the women as well recently seem to be getting more. I think people expect more from you and as soon as they’re pregnant they, they turn to you first for everything, you know, even if it’s unrelated to the pregnancy...” [Sally]	Subtheme 1: Midwives experience increasing demands and expectations	Theme 1: The midwifery profession has evolved with consequences for current practice.
“… autonomous practice has been eroded by tick boxes, litigation, umm, government controlling saying the women should have these rights, this should be happening, there is not enough midwives and all that and now it’s gone from a role of, umm, caregiving, judgement, umm, decision making for the women to being defensive in your caregiving in case of litigation ….” [Sue]	Subtheme 2: Midwifery practice has become defensive in nature.	

#### Theme 1: the midwifery profession has evolved with consequences for current practice

Participants reported that the profession of midwifery has changed over the years and that this extended scope of practise impacted on their ability to deliver what they perceived as quality individualised care. Two key issues (or subthemes) dominate this perception:

##### Subtheme 1: Midwives experience increasing demands and expectations

Midwives perceived being subject to increasing demands and expectations from both a professional perspective but also from pregnant women themselves. These issues result in midwives being faced with daily challenges (e.g. time, caseload, staffing, paperwork, etc.) which impact on their morale and ability to care for pregnant women.

##### Subtheme 2: Midwifery practise has become defensive in nature

Midwives demonstrated frustration with the fact that their practice has become increasingly complicated and governed by external influences. An extended scope of practice with increasing responsibilities and the resulting fear of potential litigation have caused midwives to become defensive in their practise.

#### Theme 2: the burden of responsibility

Providing physical activity advice and guidance to pregnant women was experienced by midwives as a disconcerting obligation where the boundaries of responsibility were blurred between health professionals, exercise professionals, and the pregnant women themselves. This perception is represented by four scenarios (or subthemes; see Table 2).

**Table 2:** The burden of responsibility

Raw data example	Subtheme	Theme
“I do, I think, umm, we are ideally placed to give that information particularly at the booking appointment; we’ve got the time capacity to do it. But I don’t think we’re fully, umm, equipped with the information really. Umm, and like he said, you know, you mentioned earlier, there are women that do, you know, variable things, you know, various things and they come to us for that information and I haven’t got the answer.” [Lucy]	Subtheme 1: Midwives are ideally placed to provide physical activity advice and guidance.	Theme 2: The burden of responsibility.
“Sometimes it’s the women’s perception, because they’ve got to take responsibility themselves at some point, but they seem to think the midwife will do it all. The midwife to do all their appointments, the midwife will get this, the midwife will do that, and it’s, it’s difficult for them to understand that we can signpost, we can give information or we can say yes or no, which is best, but it’s up to them to actually go to the stop smoking course, go to the, umm, Children’s Centres and learn about diet and things like that, and a lot of them what might be a 5 minute chat when I think I’m giving information about how good the benefits is to walk or swim, they might not, may not even perceive that as anything to do with exercise.” [Sue]	Subtheme 2: Pregnant women fail to take responsibility for themselves.	
“… I wouldn’t really advise them personally on anything, because I don’t know what is available and a lot of the, the women have come back and said, my gym instructor said ask the midwife, where I might have said to them you need to tell him you’re pregnant and they will tailor what’s suitable for you. But they don’t, they refer them back to us, so for me that’s quite a challenge on what is available out there for them when they’re pregnant.” [Tina]	Subtheme 3: Passing the buck between exercise and health professionals.	
“I, I think there should be preconception clinics, ideally, and that’s where you start the lifestyle change prior to the pregnancy, you know, and umm, talk to women about exercise then. And I think women who are wanting to conceive are going to do everything to make themselves healthy cause they want the baby. And I know you’ve got women who don’t plan pregnancies, you know, but I think if you could have, you know, ‘Well Women’ clinics up and running, you know.” [Anne]	Subtheme 4: Lifestyle change should start with preconception clinics	

##### Subtheme 1: Midwives are ideally placed to provide physical activity advice and guidance

Midwives agreed that pregnant women did not receive adequate information about physical activity during pregnancy and that the topic was not emphasised enough. However, despite feeling that they were not equipped to provide effective advice and guidance most midwives believed they were ideally placed to do so.

##### Subtheme 2: pregnant women fail to take responsibility for themselves

Some midwives also highlighted the perception that pregnant women have to take responsibility for their own health and wellbeing and that the expectation for midwives to know everything was unrealistic.

##### Subtheme 3: passing the buck between exercise and health professionals

Midwives shared their experiences and frustrations of the buck being passed between exercise and health professionals. This reluctance of professionals to accept responsibility, however, appears to affect predominantly pregnant women who are regular exercisers, often leaving them without clear guidance and support to enable continued engagement with physical activity.

##### Subtheme 4: Lifestyle change should start with preconception clinics.

Some midwives voiced the opinion that pregnancy was too late to bring about change in pregnant women’s lifestyle behaviours. They suggested that health issues should be addressed prior to pregnancy with the aim of assisting women in achieving optimum health before becoming pregnant.

#### Theme 3: providing physical activity advice and guidance is a tick box exercise

Midwives reported that the physical activity advice and guidance provided to pregnant women is limited to the initial booking appointment. Typical advice is in line with the Royal College of Obstetricians and Gynaecologists (RCOG, 2006) position statement, that is, pregnant women can continue with activities that their body is used to [9]. Three scenarios (or subthemes) are evident in relation to this theme (see Table 3).

**Table 3:** Providing physical activity advice and guidance is a tick box exercise

Raw data example	Subtheme	Theme
“To be fair, I’m sure my colleagues will say the same thing, we asked them about exercise and we have a little tick box which we do during the booking, umm, the initial booking, umm, appointments, however, we don’t kind of explore that any further.” [Lucy]	Subtheme 1: Physical activity is only addressed at the booking appointment and not revisited again unless asked about.	Theme 3: Providing physical activity advice and guidance is a tick box exercise.
“But I don’t think a pregnant woman is going to start exercising because she is pregnant necessarily. I think it is more women who already exercises, you know, who will continue and those are the ones who are seeking the advice.” [Anne]	Subtheme 2: Only pregnant women who are regular exercisers will enquire about physical activity.	
“Umm, so I think it’s more on, I don’t know if it is from area or individual midwives, but there is overall a lack and when I talk to the girls in the unit about, umm, their bookings, umm, I think everyone seems to focus on a different area. Umm, my work before was screening so I do screening, but no one seems to focus really on exercise.” [Sue]	Subtheme 3: Emphasis on physical activity is inconsistent with midwives pushing different agendas.	

##### Subtheme 1: physical activity is only addressed at the booking appointment and not revisited again unless asked about

Midwives described the typical process by which pregnant women receive physical activity advice and guidance as a tick box exercise at the booking appointment and further revealed that the topic was not explored or revisited thereafter unless it is brought up by the pregnant women themselves.

##### Subtheme 2: only pregnant women who are regular exercisers will enquire about physical activity

Participating midwives recognise that it is typically pregnant women who are regular exercisers at the time of becoming pregnant that will make enquiries as to whether they can continue whereas those who do not exercise regularly will often make excuses for not doing so and the issue is then not explored any further.

##### Subtheme 3: emphasis on physical activity is inconsistent with midwives pushing different agendas

The emphasis that is put on physical activity varies as it appears that midwives are pushing different agendas depending on their area of interest or speciality. Further, due to the amount of information that needs to be discussed and the time available, midwives report having to prioritise what they consider as relevant and important to each pregnant woman.

### What are the barriers perceived by midwives in providing effective physical activity advice and guidance to pregnant women?

Notwithstanding the value of physical activity during pregnancy, midwives felt constrained by several perceived barriers in providing effective physical activity advice and guidance. Five themes exemplify these barriers (see Table 4).

**Table 4:** Midwives’ perceived barriers in providing effective physical activity advice and guidance

Raw data	Theme
“… it’s just not having that information and, you know, potentially having to ask her to go somewhere else to get it. You know, she looks upon you as the fount of all knowledge and actually you, but that’s the thing about midwifery as well though; we don’t, we don’t, umm, we don’t claim to know everything in which case then we refer to somebody else. But actually with regard to your, you know, the exercise, it would be handy to have something in place to say well it is ok to do Zumba, it is ok to do your horse riding and we quite often have somebody who says they horse ride.” [Lucy]	Theme 1: Lack of training, knowledge and confidence.
“Time, time is a huge one. Umm, my case load is too big for a part-timer, but because of staffing levels, it’s just the way it rolls at the moment. Umm, I feel my biggest challenge at the moment is maintaining my safety. Umm, I think there’s things that I forget now, because I’ve got too much other stuff going on.” [Louise]	Theme 2: Time constraints and ensuing compromises.
“… I can’t think of any Trust or NHS leaflets specifically about exercise where there is on diet, there is on drinking, there’s on smoking, but I can’t think, except the postnatal exercises they go home with that no one ever reads.” [Sue]	Theme 3: Unawareness of suitable resources and opportunities.
“I do agree that we’re not equipped enough to formally give information about exercise, but a lot of it is common sense.” [Sue] “I do feel there is limited information, you know, and I don’t have lots of knowledge about what advice to give women, I just draw on, like my personal experience and sort of what I’ve picked up going along, you know.” [Anne]	Theme 4: Reliance on common sense and own experience as opposed to evidence-based practice
“Uh, no, I think probably most health professionals know that it’s, you know, it’s the right thing to do, umm, but I think the fear, it’s the fear attached to it, of not making sure that you’re giving the right information and umm, you know, you know, not sort of upsetting the women or you know offending the women or, you know.” [Amy]	Theme 5: Perceptions of vulnerability relating to inherent fears and exposure to risk.

#### Theme 1: lack of training, knowledge, and confidence

Lack of training, knowledge, and confidence was cited throughout as a reason for not being able to provide effective physical activity guidance and advice. Midwives were unsure if the curriculum had changed to include the topic within the public health module of current student midwives’ training but reported that it was not included as part of the training they had received. They also appeared to have difficulty in providing advice regarding specific activities, e.g. Zumba, horse riding, etc. The lack of knowledge in this area also resulted in a lack of confidence meaning that only basic advice could be provided to pregnant women.

#### Theme 2: time constraints and ensuing compromises

Midwives identify time constraints as one of the most significant barriers in providing effective physical activity advice and guidance. Typically, advice is basic and not revisited again as information and tasks are often prioritised in terms of perceived relevance and importance. In addition, having to manage time and extended caseloads means that midwives feel exposed to greater risk.

#### Theme 3: unawareness of suitable resources and opportunities

In general, midwives were unaware of suitable professional resources (leaflets, websites, books, etc.) to draw upon themselves. In most cases, midwives were happy to signpost to the NHS Choices website but felt ill-equipped to deliver tailored content. Additionally, midwives also showed a lack of awareness of suitable exercise opportunities in the local community.

#### Theme 4: reliance on common sense and own experience as opposed to evidence-based practice

A lack of training, knowledge and access to professional resources have resulted in midwives having to rely on common sense and their own experience to advise and guide pregnant women. A reliance on general as opposed to professional knowledge and resources leaves midwives questioning their authority in providing physical activity advice and guidance. However, midwives expressed the desire to be able to provide reliable and current information as part of evidence-based practise.

#### Theme 5: perceptions of vulnerability relating to inherent fears and exposure to risk

Midwives perceive being under increasing pressure to take on more responsibilities, however, due to time constraints, lower staffing levels, and increasing caseloads there is also the added pressure of having to manage risk in terms of safety and liability. When it comes to advising pregnant women regarding physical activity, midwives also fear not giving the right information, disappointing, upsetting, or potentially offending pregnant women.

### Midwives’ perceived opportunities in changing pregnant women’s physical activity behaviour

Based on their experiences of working with pregnant women in the local community, midwives identified what they perceived as feasible opportunities in changing pregnant women’s physical activity behaviour. These suggestions are illustrated through eight broad themes (see Table 5).

**Table 5:** Midwives’ perceived opportunities in changing pregnant women’s exercise behaviour

Raw data example	Theme
“The only exercise, there’s only a small amount of women who would take up anything remotely official, exercise like swimming or yoga or gym and that’s the ladies who have good jobs or husbands with good jobs, they only have one child at home, perhaps two maximum, those who can afford the time and the money.” [Kate]	Theme 1: Recognising and addressing barriers in the uptake and maintenance of exercise participation during pregnancy.
“You could do it as students, yes involve it into the uni, but as a qualified midwife we have two mandatory days a year. One of them we have smoking updates, about smoking, we have mental health updates, maybe that should be in as a separate topic for us as midwives to be promoted, or even maybe not on the generalised one but the community update, cause the community midwives are the ones that get up from the beginning. So maybe on that study day they should be involving, explaining us and finding out and giving us that information on what is out there and how we can promote that.” [Tina]	Theme 2: Professional development
“I think, umm, midwives can do the initial umm, talk, because it isn’t rocket science, everyone can give a little talk on the importance of, uh, exercise and as you said incorporate it with, it’s walking, or perhaps taking the children swimming, playing in the park, that sort of thing. But it would be nice to have something then we can say, if you like to know more about this, this is where you go to or if you’re happy, like with smoking, it’s filling out a form and send it off and then someone will contact them.” [Sue]	Theme 3: Inter-professional collaboration
“No, I think maybe the only other thing is that maybe some kind of online, umm, facility for women, or an app. I mean I know there are loads of apps out there, umm, but we don’t have any real way of, you know, checking whether they’re credible or not, so maybe some kind of NHS approved app that could motivate women in pregnancy specifically.” [Vicky]	Theme 4: Communicating effectively through simple, credible resources
“It would be good, it would be really nice actually if, you know, especially places like Children’s Centres could put on an exercise facility for pregnant women and some of the midwives could be trained to deliver that.” [Sally]	Theme 5: Improved access, availability and awareness of suitable activities in the local community.
“And I think actually it would be really good because then it gives women the chance to meet other women that are pregnant as well so it would really, it would really, umm, help to incorporate some women into groups that find it very difficult to come to groups, you know, if they’re going to do an exercise class then they get to meet people every week at the same class.” “Umm, so they would build up a social network as well which is really important.” [Sally]	Theme 6: Encouraging a support network
“I would say that, umm, like most things for pregnant women, you feel that you have to sell it, umm, and like the stop smoking, like all of the health things, umm, I would probably say it will make your birth easier and I would say that umm it will help with not gaining so much weight in your pregnancy, that that’s not necessary and that you don’t want to pile it on in pregnancy and they’re the things that would help if you do some, you know, small amounts of exercise.” [Amy]	Theme 7: “Selling” physical activity by challenging misconceptions and focusing on benefits.
“And for lots of ladies that’s quite good, rather than from what I’ve seen in the past, umm, they start off and they will fizzle out after a while, but if they’ve got, knowing that perhaps they can get free children’s places or, umm, I don’t know, whatever vouchers to use, umm, that will keep them going more.” [Sue]	Theme 8: Suitable motivation, incentives, and reward.

#### Theme 1: Recognising and addressing barriers in the uptake and maintenance of physical activity during pregnancy

Midwives recognised potential barriers (e.g. time, childcare, finances, work commitments, etc.) that could prevent women from engaging in physical activities during pregnancy. They then also made suggestions as to how these barriers could be addressed in order to limit reasons for not attending and improving the uptake and maintenance of physical activities. For example, activities should be free at the point of access and a crèche should be provided.

#### Theme 2: professional development

Having identified lack of knowledge and training as a potential barrier in providing effective exercise advice and guidance, midwives suggested that training could be facilitated through one of their mandatory study days or as an optional online Continuous Professional Development (CPD) activity. Some midwives also suggested that physical activity advice and guidance should be incorporated as part of the initial training that student midwives receive. A final suggestion included assigning the remit of physical activity to a midwife (i.e. specialist lead midwife or physical activity champion) who could lead on the topic and update other community midwives with resources and current evidence.

#### Theme 3: inter-professional collaboration

Midwives had differing views regarding an exercise referral service for pregnant women. Some suggested that midwives could do the initial consultation and then refer on to a separate service, which could involve specialist midwives or health trainers. Other midwives were more sceptical about uptake and suggested that a collaborative service provided during clinic sessions would be more successful.

#### Theme 4: communicating effectively through simple, credible resources

Acknowledging that they were unaware of suitable professional resources to draw on, midwives suggested the need for simple and reliable resources (e.g. bullet style leaflets) to support their practice. Furthermore, it was also suggested that an app or online facility that was endorsed by the NHS could be a useful educational and motivational tool.

#### Theme 5: improved access, availability, and awareness of suitable activities in the local community

Having identified that they were unaware of the activities available, midwives suggested that Children’s Centres are ideally located within the local community to facilitate activities for pregnant women. They were also keen to support services hosted by appropriately trained professionals or where midwives received additional training to host activity sessions themselves. Furthermore, midwives also suggested that physical activity advice and guidance should be specific to a pregnant women’s environment and that this could include everyday activities such as walking and taking care of a family.

#### Theme 6: encouraging a support network

Midwives were keen to point out and support the potential social benefit of group activities which could also serve the purpose of providing a support network throughout and following pregnancy. They further recognised that engaging the pregnant women’s partner could be beneficial in terms of the support and motivation they can provide.

#### Theme 7: “selling” physical activity by challenging misconceptions and focusing on benefits

When asked, midwives were able to recognise the advantages and disadvantages of an active pregnancy as identified and perceived by pregnant women. However, midwives were divided in their opinion as to whether behaviour change campaigns should be focused on potential benefits to either mum or baby. Participating midwives recognised that pregnant women may experience various concerns and challenges and felt the need to “sell” the idea of physical activity by challenging misconceptions and focusing on benefits.

#### Theme 8: suitable motivation, incentives and reward

Most midwives believed that internal motivation as opposed to external rewards would be more effective in achieving long-term behaviour change. Whilst some midwives felt no incentives should be offered, others were of the opinion that a reward or incentive should be used to attract pregnant women to join activities in the first instance. Midwives also suggested that incentives such as childcare vouchers could be used to address some of the barriers that pregnant women may be faced with.

## Discussion

The purpose of this study was to gain insight into midwives’ perspectives of providing physical activity advice and guidance to pregnant women in the East Kent region of England. Interrelating themes were identified around three research questions. In discussing these findings, midwives’ views are also put in context of Better Births, a report of the National Maternity Review [13] which sets out the vision for maternity services in England. The report set out proposals with the aim of making maternity care safer and giving women greater control and more choices in relation to the care they receive. It is recognised in this report that “each women needs to engage in a relationship with her own midwife and other health professionals, acting on advice where she can make a difference, e.g., by accepting help to give up smoking, having a healthy diet and being physically active” (pp. 84–85). Triangulating the findings of this study with those of the report recently published by the National Maternity Review [13] provides a novel vantage point for the better understanding of midwives’ perspectives that might itself support the development of more effective physical activity policy and practice going forwards.

When considering their role and responsibility in providing physical activity advice and guidance, participants commented that the profession of midwifery had evolved and that the resulting vicissitudes had consequences for their current practice. Specifically, midwives experienced being subjected to increasing demands and expectations associated with an extended scope in practice. These findings echo that of the National Maternity Review [13] “with an increasing administrative burden cited as a particular difficulty. This reduced the amount of time that could be spent with women, increasing the likelihood of mistakes and missed opportunities to spot problems. A perceived litigious culture was partly to blame …” (p. 38). Similarly, midwives participating in this study perceived being exposed to greater risks. The ensuing threat of litigation and the associated costs involved have caused “midwives to practise in a risk-averse way, inhibiting their ability to support some of the choices that women may want to make, contributed to the administrative and data collection burden, and undermined multi-professional working” (p. 39) [13].

Indeed, time constraints were perceived by midwives as one of the most consuming barriers in providing effective physical activity advice and guidance. Of consequence is how pregnant women perceive the resulting lack of information. For example, Olander, Atkinson, Edmunds, and French [15] point out that due to health professionals not having enough time to discuss gestational weight gain, pregnant women interpreted this as being unimportant. A further consequence of time constraints is that information and tasks are then prioritised which infers that the topic of physical activity is often neglected and expectant mums are not being presented with key evidence-based messages allowing them to make informed decisions. The National Maternity Review [13] recognises that changes will need to be made to midwifery staffing allocations so that midwives could “have more time to be able to explain a woman’s choices and personalise the advice she receives” (p. 96).

Resonating the findings of previous research [5, 15–18], this study found that midwives provided inadequate physical activity advice and guidance. Information was basic and limited to the initial booking appointment. Issues surrounding physical activity or lack thereof was not explored or revisited later in the pregnancy unless it was brought up by the pregnant women themselves. Specifically, women who were regular exercisers at the time of the booking appointment were most likely to initiate further discussion throughout their pregnancy. This implies that inactive pregnant women receive less information than already active women. Similar to the review by Heslehurst et al. [6], the present study indicated variation in midwives’ emphasis of physical activity during pregnancy with participating midwives reporting that information had to be prioritised according to perceived importance and relevance.

Nonetheless, most midwives agreed that they were ideally placed to provide physical activity advice and guidance but that it was ultimately up to pregnant women to take responsibility for their own health and wellbeing. Whilst being in control of their care is also the desire of pregnant women, Baroness Cumberland points out that “with this control comes a responsibility which mothers must accept and professionals must support – that personal health and fitness are integral to safe and fulfilling childbearing” (p. 4) [13]. However, in acknowledging the increasing evidence that regular physical activity during pregnancy improves health outcomes for both mother and baby [2], we believe the onus is on midwives to disseminate information that will allow pregnant women to make informed choices regarding their physical activity behaviours or indeed the consequences of a sedentary lifestyle.

Midwives, however, felt that they were not equipped in providing effective physical activity advice and guidance to pregnant women. The identified lack in training, knowledge, and confidence resulted in midwives not being able to address the issues surrounding physical activity exhaustively and having to rely on common sense and their own experience to advise and guide pregnant women. Given midwives’ central positioning in the care pregnant women receive and the role they have in disseminating information, they must be provided with the opportunity to improve their knowledge and confidence [15]. This requirement appears to not be confined to a UK context, for example, Lindqvist and colleagues identified a need for additional physical activity training provision within Swedish midwifery education [18]. Although the need for greater investment in education and training is also highlighted in the National Maternity Review [13], training around physical activity and public health is not mentioned specifically. Importantly, not any of the midwives participating in this study were aware of relevant training opportunities, however, they appeared willing to improve their knowledge and proposed that training or upskilling should be facilitated on study days or as online CPD opportunities.

The National Maternity Review report [13] identifies that pregnant women have “expressed frustration over receiving conflicting advice from different healthcare professionals throughout their care” and “wanted information to be evidence-based” (p. 33). In a recent qualitative study, Evans, Walters, Liechty, and LeFevour [19] found that a lack of knowledge or receiving misinformation or unclear advice contributed to pregnant women experiencing uncertainty about the physical activities they could engage with. This uncertainty quite often resulted in the participating pregnant women having to rely on informal sources, such as the internet, as opposed to that of a health professional. However, midwives are themselves subjected to the same level of information as the general public and are unaware of additional professional resources to draw upon. Consequently, they felt unable to address the topic from a position of authority. It is thus not surprising that participating midwives also expressed the desire to be able to provide reliable and current information as part of evidence-based practice. This evidence base is becoming increasingly relevant with a current focus in literature on the positive birth and health outcomes for both mother and baby [2]. For example, meta-analytic evidence shows that regular exercise is associated with a reduced risk of gestational diabetes, a lower prevalence of excessive maternal weight gain [20], and an increased probability of a normal delivery in healthy pregnant women [21]. Similar evidence should be used to inform practice, challenge exercise related misconceptions and elaborate on potential benefits.

Midwives showed awareness of the potential social benefit of group activities aimed specifically at pregnant women and were keen to support these. However, there was a general lack of awareness and confidence in whether suitable and credible exercise opportunities existed in the local community. This implied that they were less likely to promote specific activities, such as aqua natal, but promoted general activities, such as swimming, instead. Similarly, Heslehurst and colleagues’ [6] review suggest that healthcare professionals perceived a lack in supporting physical activity services being available to pregnant women. Pregnant women have, however, expressed a desire for “locally relevant information about the services available, and for there to be time to discuss the information with a healthcare professional” (p. 33) [13].

Participating midwives recognised that engaging pregnant women’s partner could be beneficial in terms of the support and motivation they can provide. This sentiment is highlighted in the report by the National Maternity Review [13] where expecting women stated that they “relied on their partner to support them in pregnancy and with the care of the baby and the NHS needed to recognise this and help their partners to help them” (p. 33). This finding is particularly interesting in the context of De Vivo, Hulbert, Mills, and Uphill’s [22] meta-analysis showing that subjective norm, or the perceived social pressure to conform to how significant others think an individual should behave, is an important concept influencing the physical activity intentions of pregnant women.

Midwives in this study articulated their frustration with the fact that the responsibility of providing physical activity advice and guidance is often a case of passing the buck between health and exercise professionals. However, as midwives are central to the care of pregnant women and given the fact that they “must have the ability to communicate effectively with all members of the maternity team, other professionals, women receiving care and their family members” (p. 7) [23], it is therefore not unreasonable to suggest that communication and referral pathways should also include exercise professionals. Inter-professional communication, and indeed collaboration implies that knowledge and responsibility with regards to physical activity advice, guidance, and motivation will be shared in such a manner that it meets the specific needs of a pregnant woman [24]. The notion of a collaboration between healthcare and exercise professionals was also identified as an opportunity to alleviate some of the time constraints of current practise. Participating midwives explained that they had access to additional care pathways and services for various health issues (e.g. obesity, diet, smoking, alcohol, etc.), however, for exercise there was nothing in place. Some midwives suggested that they could do the initial consultation and then refer suitable pregnant women to a separate service for further advice and guidance. Other midwives were, however, more sceptical about uptake of such services and suggested that pregnant women would give it “lip service” during the appointment and then not attend. Although such a referral pathway does not currently exist, the viability and cost-effectiveness of including pregnant women in the existing exercise referral scheme [25] or a similar service should be explored. Future research should also investigate the challenges and effectiveness of inter-professional collaboration between healthcare and exercise professionals in the context of antenatal care.

It was, however, the view of some midwives in this study that pregnancy was too late to bring about change in pregnant women’s lifestyle behaviours and that health issues should be addressed in preconception clinics. This rationale is in some way reflective of current thinking involving a life-course approach to women’s health which aims to maximise every contact opportunity to improve women’s lifestyle and general health [26]. Indeed, the “women’s health network” concept advocates that “preconception care can improve maternal and newborn health by providing the foundation for a good pregnancy and birth experience”, whilst also promoting pregnancy as the “optimum time to help promote a healthy lifestyle and introduce preventative measures for reducing ill health in the mother and baby” (p. 1) [26]. Pregnancy presents multiple opportunities to influence the health and wellbeing of mothers and their children and may also be the first time that a woman and her family have continued contact with health services [13]. Whilst the proposed women’s health network is yet to materialise, maternity services are well established with opportunities to improve women’s and children health being missed far too often. Midwives’ role in securing better outcomes for mothers and babies in the broader context of population health needs to be made more explicit [27].

Communicating about health behaviours have, however, also brought to light perceived issues of vulnerability. Midwives disclosed a fear of not giving the right information, disappointing, upsetting, or potentially offending pregnant women. Similarly, Schmied, Duff, Dahlen, Mills, and Kolt [28], reported that when discussing the issue of obesity, midwives who were overweight or obese themselves had described feeling either comfortable stating that they were not good role models or uncomfortable that their body image portrayed them as not being good role models. However, midwives who were normal or underweight also felt uncomfortable broaching the subject. Likewise, Foster and Hirst [29] reported that midwives expressed concern about upsetting pregnant women as they risk spoiling the pregnancy and the relationship between them breaking down. Issues of authenticity and vulnerability are thus barriers to midwives being able to communicate effectively regarding health behaviours or as Foster and Hirst [29] reported, absolving themselves from addressing these issues entirely.

Despite facing several challenges, midwives identified eight feasible opportunities in changing pregnant women’s physical activity behaviour, which included: (1) recognising and addressing barriers in the uptake and maintenance of exercise participation during pregnancy, (2) professional development, (3) inter-professional collaboration, (4) communicating effectively through simple, reliable resources, (5) improved access, availability and awareness of suitable activities in the local community, (6) encouraging a support network, (7) “selling” physical activity by challenging misconceptions and focusing on benefits, and (8) providing suitable motivation, incentives and reward. These suggestions and the issues raised by midwives should continue to be explored in both practice and future research endeavours.

### Limitations

It is necessary to acknowledge that both the authors are exercise scientists with the opinion that women should be able to enjoy active pregnancies. This reflective position may therefore have influenced our interpretation of the data. However, our aim was to be a voice for midwives and to represent their opinions accurately. Rather than framing the study based on our own perceptions, the semi-structured interview schedule was informed by data obtained from pregnant women participating in an earlier phase of the mixed methods research project [10]. However, whilst the interviewer was portrayed as an “outsider” and it was made clear that there were no right or wrong answers, it became apparent that the study in itself raised awareness of the topic prompting participants to discuss their practises with colleagues. This is illustrated in the following quotations:

“Do you know what, now, since meeting you and, you know, and umm, and speaking to the girls after they’ve spoken to you actually *(laughing),* I realised that we don’t, umm, we don’t put enough emphasis on exercise during pregnancy, well I, I know, myself, I don’t.” [Lucy].

“Physical exercise, how could you change that? Since I’ve seen you, I talk about it more, does that help *(laughing)*.” “I do speak about it more and I notice that generally, umm, collectively as a little group of midwives we’ve spoken about exercise more, cause we’ve all gone ‘what do you do to promote’ and it will be, it’s quite interesting of what we all say and do …” [Louise].

Although discussions between colleagues may have resulted in introspection and influenced the interview discourse, there is no reason to believe that midwives were not sincere in their accounts or that this affected the study’s outcome.

## Conclusion

Midwives feel strained by the increasing demands of their profession and pregnant women’s expectation for them to be the “fount of all knowledge” to who they turn first for all information. This perception includes their role and responsibility in securing better health outcomes for mothers and babies through the promotion of physical activity. However, several barriers exist which prevent effective physical activity advice and guidance from being delivered with authority. Whilst hypothetically midwives are “ideally placed” to advise regarding physical activity, this is not always feasible. Instead, they may be better placed as agents in a wider network of facilitators that share a consistent and confident message regarding physical activity during pregnancy. This notion has far reaching implications for practice, policy, research thereby facilitating the normalisation of active pregnancies in the wider population.

## Supplementary information

**Additional file 1.** Interview schedule.

## Data Availability

Further data concerning the mixed methods multiphase research project (e.g. methodology, first author’s bias, reflexivity, etc.) and additional data (e.g. participant quotes, information sheet, consent form, demographics questionnaire, data analysis, etc.) pertaining to this transcript is available in the CReATE repository, https://create.canterbury.ac.uk/15881/2/De%20Vivo.pdf Interview transcripts will not be made available to protect participants’ anonymity.

## Abbreviations

NHS: National Health Service
RCOG: Royal College of Obstetricians and Gynaecologists
