# Supplementary material for: “They turn to you first for everything”: insights into midwives’ perspectives of providing physical activity advice and guidance to pregnant women
Source: BMC Pregnancy Childbirth. 2019 Dec 3;19:462. doi: 10.1186/s12884-019-2607-x (PMC6891952; doi:10.1186/s12884-019-2607-x)
Supplement: Supplementary file 1 — Additional file 1. Interview schedule. [file 12884_2019_2607_MOESM1_ESM.pdf]

Canterbury Christ Church University

SEMI-STRUCTURED INTERVIEW SCHEDULE

Section 1: Introductory Questions

1. Could you begin by telling me why you wanted to be a midwife and how you got started in the profession?
2. What do you see as the main roles and responsibilities of being a midwife?
3. Can you, in a few words, sum up what you see as the main challenges of your profession but also what you particularly enjoy about it?

Section 2: Exercise and Pregnancy Questions

Definition of exercise:
Exercise is defined as any regular moderate physical activity that requires you to expend energy. For example, performing any one or a combination of the following activities on most days of the week: walking 2 miles in 30 minutes, gardening for 30-45 minutes, pushing a stroller 1.5 miles in 30 minutes, washing floors/windows for 45-60 minutes, swimming for 20 minutes, dancing for 30 minutes, running 1.5 miles in 15 minutes, etc.

1. We asked pregnant women what they thought the main advantages and disadvantages of exercise during pregnancy were. What do you think they said and what would you have said these were from a midwife's perspective?
2. Do you think that pregnant women receive adequate information regarding exercise during pregnancy?
3. What is your opinion regarding physical activity during pregnancy? How important do you think it is in the grand scheme of things?
4. How confident do you feel providing advice about exercise during pregnancy?
5. Do you feel that your training has adequately prepared you for providing exercise advice?
6. How do you personally feel about physical activity and do you think that this has in any way influenced your professional practise?
7. Have you ever encountered a particularly challenging question or situation with regards to exercise during pregnancy?
8. I'm going to read you an extract from a published article and I would be interested in you your thoughts.

"One of the most important issues arising from this study was the perceived lack of accessible information and advice on the benefits of physical activity during pregnancy. Midwives were viewed as being ideally placed to advise and support women about physical activity in pregnancy. However, many of the study participants described how their midwives had not given them any advice or guidance on physical activity. It has also been suggested from the findings of previous research that healthcare providers are often reluctant to advise on diet and activity changes during pregnancy" (Weir et al., 2010, p. 6).

Do you agree with this? Would you say that this is a fair reflection of current practise?

9. What are the most significant barriers to you promoting an active pregnancy?
10. When it comes to giving advice about exercise during pregnancy, which resources can you draw on or make use of (e.g. have you referred pregnant women to any online resources)? Is there a reason for choosing these resources?
11. How do you keep up-to-date about the information that is available for pregnant women?
12. Do you think that more should be done to raise awareness about the guidelines for exercise during and after pregnancy? If so, what suggestions do you have and where do you think the focus should be e.g. birth outcomes, benefits for mum, benefits for baby, etc.?
13. Do you think that more should be done to provide opportunities for pregnant women to be active? For example:
    - Do you think that pregnant women should have access to supervised training facilities and/or opportunities?
    - Do you think that pregnant women should have access to free training facilities and/or opportunities?
    - Do you think that pregnant women should be incentivised to adopt a more active pregnancy?

Section 3: Concluding Questions

With regards to exercise during pregnancy, are there any additional issues that we've not talked about, that you think would be important to address?

Interview Prompts/Probes:

- You haven't mentioned X···
- You've said/mentioned Y···
- What do you mean by Z?
- Would you elaborate on that?
- Could you say some more about that?
- That's helpful. I'd appreciate if you could give me more detail.
- I'm beginning to see the picture but some more examples might help.
- What you're saying now is very important, and I want to make sure that I understand exactly what you mean by it, please explain some more.
